# Psychological distress and resilience in patients with gastroenteropancreatic neuroendocrine tumor

**DOI:** 10.3389/fendo.2022.947998

**Published:** 2022-11-16

**Authors:** Lili Song, Yanshuo Cao, Jie Li, Ming Lu, Lili Tang

**Affiliations:** ^1^ Department of Psycho-Oncology, Key Laboratory of Carcinogenesis and Translational Research (Ministry of Education/Beijing), Peking University Cancer Hospital and Institute, Beijing, China; ^2^ Department of Gastrointestinal Oncology, Key Laboratory of Carcinogenesis and Translational Research (Ministry of Education/Beijing), Peking University Cancer Hospital and Institute, Beijing, China

**Keywords:** neuroendocrine tumors, psychological distress, resilience, anxiety, depression, coping

## Abstract

An increased incidence of gastroenteropancreatic neuroendocrine tumors (GEP-NETs) has been reported in many countries. However, the prevalence and impact factors of psychological distress and resilience in patients with GEP-NETs are unclear. We recruited 200 patients with GEP-NETs to assess psychological distress and resilience. Measures comprised the Distress Thermometer, the Hospital Anxiety and Depression Scale, Connor-Davidson Resilience scale and Medical Coping Modes Questionnaire. Our results found that the prevalence of distress, anxiety, depression and low resilience were 31.5%, 31%, 17.8%, and 25.9%, respectively. Female patients were more likely to be distressed, as were those with NET Grade 1, were partly aware of diagnosis, and had known the diagnosis less than 3 months. Distress positively correlated with acceptance-resignation, and resilience positively correlated with confrontation and avoidance. Resilience negatively correlated with psychological distress. Patients coping disease with acceptance-resignation had higher odds of anxiety, depression, and low resilience. Our findings indicate that psychological distress and low resilience were common in patients with GEP-NETs. This suggests a need to integrate psychosocial domain into GEP-NETs clinical practice.

## Introduction

Gastroenteropancreatic neuroendocrine tumors (GEP-NETs) are recognized as the most common type of NETs, and are originated from gastrointestinal tract and pancreatic neuroendocrine cells (1). GEP-NETs have been considered as a rare type of diseases with an incidence of 3.56-5.25 per 100,000 (1, 2). However, an increased incidence of GEP-NETs has been reported in many countries (1–3), and GEP-NETs have gained significant attention in China (4, 5). GEP-NETs are frequently associated with unique epidemiological and biological features, which lead to specific medical problems and sources of psychosocial distress, such as anxiety and depression (6–8). A high rate of depression symptoms among GEP-NET patients has been reported, ranging between 26% and 40% (7, 8). Meanwhile, several studies indicated that patients with GEP-NETs had low level of depression, about 13% (9, 10). Previous studies have also reported varying rates of anxiety in GEP-NET patients, ranging from low (19%) to high (33%) (7–10). Worries about the prognosis of the disease, the family’s situation, tests, and examinations were frequently reported in GEP-NET patients (7). However, similar studies on psychosocial distress of Chinese GEP-NETs patients are lacking. As China’s cultural background has its uniqueness, especially in facing diagnosis of malignant tumors, it is important to better understand GEP-NETs patients’ mental health in China. Family members play an important role in treatment related decision-making process in China, and most oncologists would first inform and discuss a malignant tumor diagnosis with a patient’ family members instead of the patient (11). One study showed cancer patients not knowing the diagnosis reported higher level of depression in Romania (12), but whether this is also true in China is unknown.

Studies have highlighted a number of unmet needs including psychological needs and diagnosis of mental health in neuroendocrine tumor (NET) patients. All groups of participants, including patients, advocates, and health care professionals, believed NETs patients’ psychological care needs were often not met or not met at all (13). Research showed that adopting a patient inclusive multidisciplinary team (MDT) could increase patients’ satisfaction with care. However, even provided with patient inclusive MDT, 14% of patients reported not having enough contact (13). Other findings demonstrated that higher satisfaction with physicians’ care was related to lower levels of anxiety and depression in NET patients (14). These results indicated that MDT strategies, especially the psychological support, for NET patients needed to be optimized.

Despite the tremendous psychosocial distress associated with GEP-NETs diagnosis and treatment, some GEP-NETs patients showed good adaptability to their diagnosis and treatment using coping strategies and social support (15). The other strategies cancer patients often used include optimism, hope, spirituality, resilience, and posttraumatic growth (16). Resilience has been defined as “an individual’s ability to maintain or restore relatively stable psychological and physical functioning when confronted with stressful life events and adversities” (16). A growing body of literature has examined the association between resilience and optimism, hope, spirituality, posttraumatic growth, adaptive coping strategies, less anxiety and depression, better mental health and treatment outcomes in cancer patients (16–19). For instance, in a study investigating resilience in hematopoietic cell transplantation survivors, patients reporting low resilience scores had higher odds of having psychological distress and being in the lowest quartile for mental health–related quality of life (17). Although resilience was initially considered to be a psychological variable, it has gradually become a psychological indicator and may contribute to improving psychological distress (18, 19). Therefore, resilience-fostering interventions to nurture optimism, hope, and spirituality could help patients trying to fight cancer (16). However, there is no data available on resilience in GEP-NETs patients and it is essential for evaluation of resilience in GEP-NETs patients to better understand patients’ psychological status.

As the epidemiological and biological features of GEP-NETs are unique, and studies about psychological care for patients with GEP-NETs are relatively sparse and with varied results, understanding psychological status of this group of patients and factors associated with psychological distress and resilience would contribute to psychological wellbeing in patients with GEP-NETs. The purposes of this study included: 1. to evaluate the levels of psychological distress and resilience in patients diagnosed with GEP-NETs in a tertiary hospital in China; 2. to identify factors associated with psychological distress and resilience in patients with GEP-NETs in a tertiary hospital in China; 3. to give directions of optimization of psychological management of GEP-NETs patients.

## Materials and methods

### Inclusion and exclusion criteria

For this cross-sectional study, patients were recruited by convenience from August 2017 to December 2019 who received treatments at the Gastrointestinal Oncology Department of Peking University Cancer Hospital in Beijing, China. Major inclusion criteria include: age 18 years or older; had confirmed pathological diagnosis of well differentiated GEP-NETs G1 or G2 according to the 2017 World Health Organization (WHO) classification of Tumours (20); willing to participate the study. Major exclusion criteria include: patient had a cognitive impairment or a disturbance of consciousness; patient was complicated with serious complications such as other malignancies or dyscrasia; NET G3 or neuroendocrine carcinoma (NEC, according to the 2017 WHO Classification of Tumours) was excluded.

This study was approved by Ethics Committee of Peking University Cancer Hospital ethics committee (Reference number: 2017KT43) and was conducted strictly in accordance with the ethical requirements in good clinical practice, and strictly in compliance with the Declaration of Helsinki. All patients provided written informed consent (ICF) before enrollment.

### Assessment

Sociodemographic information (age, sex, marital status, education, work-status, income and awareness of diagnosis) was collected using standard questionnaires. Medical information (tumor site, tumor grade, metastasis, and time span after diagnosis) was obtained from each patient’s clinical chart.

Psychological distress was measured using the Distress Thermometer (DT) and the Hospital Anxiety and Depression Scale (HADS). The DT consists of a single-item 11-point self-report scale ranging from “No distress” (0) to “Extreme distress” (10, 21). The DT has been validated as a screening tool for Chinese cancer patients; a DT score of 4 is the optimal cutoff value (22).

The HADS is a 14-item self-report instrument comprising two subscales: Anxiety (HADS-A) and Depression (HADS-D) (23). Both subscales consist of seven items, and the scores range from 0 to 21. For both subscales, higher scores reflect more anxiety and depressive symptoms. The Chinese version of the HADS has shown good reliability and validity (24). Scores of 9 or more indicate possible cases of depression or anxiety (25).

Resilience was measured with the 25-item Connor-Davidson Resilience scale (CD-RISC) (26). CD-RISC is rated on a 5-point Likert scale ranging from 0 (not true at all) to 5 (true nearly all of the time). The total score ranges from 0 to 100, with higher scores indicating greater resilience. CD-RISC has been well validated in Chinese populations (27).

Coping strategy was assessed by the Medical Coping Modes Questionnaire (MCMQ) (28). This questionnaire is widely used in China for assessment of patients’ coping strategy (29). It contains 20 items and assesses three illness-related coping modes: Confrontation, Avoidance and Acceptance-Resignation. Each item is scored on a four-point Likert scale ranging from 1 (never) to 4 (always). Scores from the three subscales were calculated, and a higher score indicates a higher probability of using the corresponding coping strategy.

### Statistical analysis

Descriptive statistics were used to assess the sample characteristics and the distribution of the studied variables. Reliability of the instruments was tested with Cronbach’s Alpha coefficient and found to be α=0.800 for HADS-A, α=0.728 for HADS-D, α=0.729 for Confrontation, α=0.561 for Avoidance, α=0.777 for Acceptance-resignation and α= 0.958 for CD-RISC. Data were first tested for normality and homogeneity of variances. Because the data did not follow a normal distribution, Mann–Whitney U‐test or Kruskal–Wallis nonparametric test were used to analyze the differences in distress and resilience of the samples with different demographics and medical characteristics. Bivariate analyses were performed between demographic variables, clinical variables, and psychological variables. Logistic regression analyses were used to identify factors influencing the level of distress and resilience. We considered high or low resilience (defined at the upper or lower CD-RISC quartiles, respectively). DT, HADS, and CD-RISC were dichotomized at < 4/≥4, < 9/≥ 9 and < 50/≥ 74, respectively. All significant variables were entered in the regression analysis at *p* <.05 and removed from the model at *p* >.10. Statistical analyses were conducted using SPSS version 22.0 (IBM Corporation, Armonk, NY, USA). Statistical significance for all tests was set at 0.05 or less.

## Results

### Participants’ characteristics

In this study, a total of 224 potential participants were screened, and 200 patients were recruited. The primary reasons for declining were lack of interest (n = 14) and no enough time filling the questionnaires (n = 10). Three incomplete questionnaires were excluded because of missing data. Finally, data from 197 questionnaires were extracted and analyzed.

Participants (n = 197) were predominantly middle-aged or elderly, male, married, and educated to high school or above (**Table 1**). The most common primary sites are pancreas (46.2%), followed by rectum (21.8%), liver (11.2%), small intestine and colon (10.7%), and stomach (10.2%). The pathological diagnosis of most patients (86.8%) was NET G2 and 85.8% had metastatic disease. More than half of the patients (50.8%) partly understood the diagnosis. Partly understanding the diagnosis means patients don’t know the exact prognosis due to patients are not willing to know the truth or patient’s family members don’t want patient to know. Median span of time from diagnosis to our interview was 7 months (range 0.23-188), and 63.5% of span of time were more than 3 months.

**Table 1:** Demographic and clinical characteristics of participants (N = 197).

Variable	N	%	DT	Z/*χ^2^* (P)	CD-RISC	Z/*χ^2^* (P)
			Median (Range)		Median (Range)	
Age (years)						
≤44	51	25.9	2 (0,9)	−0.58 (.565)	64 (0,92)	−0.51 (.607)
>44	146	74.1	2 (0,10)		65 (0,92)	
Sex						
Male	109	55.3	2 (0,9)	−2.21 (.027*)	61 (18,92)	−2.11 (.035*)
Female	88	44.7	3 (0,10)		67 (0,92)	
Marital status						
Single/divorced/widowed	23	11.7	3 (0,8)	−1.78 (.076)	57 (35,92)	−1.96 (.050)
Married	174	88.3	2 (0,10)		65 (0,92)	
Education						
≤ 9 years	67	34	2 (0,10)	−1.09 (.274)	61 (0,92)	−2.39 (.017*)
> 9 years	130	66	2 (0,9)		67 (18,92)	
Work−status						
employed	67	34.0	2 (0,9)	0.58 (.749)	68 (0,92)	9.26 (.010*)
Retired	64	32.5	3 (0,9)		65 (4,92)	
Unemployed	66	33.5	2 (0,10)		65 (0,92)	
Income (Yuan per month)						
≤3000	73	37.1	2 (0,9)	0.11 (.945)	61 (0,88)	4.87 (.088)
3000 −5000	79	40.1	2 (0,10)		67 (0,92)	
≥ 5000	45	22.8	2 (0,9)		65 (0,92)	
Site of primary						
pancreas	91	46.2	2 (0,9)	1.07 (.899)	64 (27,92)	6.49 (.165)
rectum	43	21.8	2 (0,9)		67 (0,92)	
liver	22	11.2	2 (0,6)		69 (37,83)	
small intestine and colon	21	10.7	2 (0,8)		55 (0,90)	
stomach	20	10.2	2 (0,10)		64 (0,90)	
Tumor grade^§^						
Grade 1	26	13.2	3 (0,10)	−2.15 (.031*)	66.5 (4,88)	−0.41 (.682)
Grade 2	171	86.8	2 (0,9)		64 (0,92)	
Metastasis						
No	28	14.2	3 (0,10)	−1.74 (.082)	65.5 (4,88)	−0.29 (.772)
Yes	169	85.8	2 (0,9)		64 (0,92)	
Awareness of diagnosis						
Fully aware of ^a^	88	44.7	1.5 (0,9)	6.46 (.040*)	68 (0,92)	8.55 (.014*)
limited aware of diagnosis ^b^	100	50.8	3 (0,10)		59.5 (0,92)	
unaware of diagnosis ^c^	9	4.6	1 (0,8)		64 (35,85)	
Time span from diagnosis (months)						
≤3	72	36.5	3 (0,10)	−2.25 (.024*)	62 (0,92)	−0.82 (.414)
> 3	125	63.5	2 (0,9)		65 (0,92)	

^a^Patients knew the name and stage of the disease, treatment plan, and prognosis.

^b^Patients knew the name and stage of the disease, treatment plan, without knowledge of prognosis.

^c^Patients did not know any information regarding their diagnosis.

^§^According to the 2017 WHO Classification of Tumours. CD-RISC, Connor-Davidson Resilience Scale; DT, Distress Thermometer. *p < .05.

### Prevalence or scores of psychological variables

The prevalence of distress, anxiety and depression in GEP-NETs was 31.5% (62/197), 31% (61/197), and 17.8% (35/197), respectively. The prevalence of low and high resilience was 25.9% (51/197) and 25.4% (50/197), respectively.

The lower quartile, median, and upper quartile distress scores were 0, 2, and 4, respectively (**Table 2**). The lower quartile, median, and upper quartile resilience scores were 50, 65, and 74, respectively. The median Confrontation, Avoidance and Acceptance-resignation scores were 19 (range 9-28), 17 (range 10-25), and 9 (range 5-18), respectively.

**Table 2:** Relationships between distress, resilience and coping strategies, depression, and anxiety.

Variable	Median (Range)	DT		CD-RISC	
		r	p	r	p
Confrontation	19 (9,28)	0.00	.967	0.20	.006*
Avoidance	17 (10,25)	-0.11	.110	0.31	<.001*
Acceptance-resignation	9 (5,18)	0.40	<.001*	-0.39	<.001*
HADS-A	7 (0, 16)	0.56	<.001*	-0.41	<.001*
HADS-D	5 (0, 15)	0.40	<.001*	-0.43	<.001*
DT	2 (0,10)			-0.29	<.001*
CD-RISC	65 (0,92)	-0.29	.000		

CD-RISC, Connor-Davidson Resilience Scale; DT, Distress Thermometer; HADS-A, Hospital Anxiety and Depression Scale-Anxiety; HADS-D, Hospital Anxiety and Depression Scale-Depression.

*p<.05.

### Differences in distress and resilience by demographics and medical characteristics

Sex, tumor grade, knowing of diagnosis and time span after diagnosis were associated with distress score (**Table 1**). The median distress score among female patients was 3 compared to 2 among male patients (*p* <.05). The median distress score among patients with NET G1 was 3 compared to 2 among those with NET G2 (*p* <.05). Participants who partly knew diagnosis reported median distress score of 3 compared to 1 or 1.5 among those who knew nothing or were fully aware of the diagnosis (*p* <.05). Participants whose time span from diagnosis to interview were less than 3 months reported a median distress score of 3, compared to 2 among those with a time span of more than 3 months (*p* <.05).

Sex, education, work-status and knowing of diagnosis were associated with resilience scores (**Table 1**). The median resilience score among female patients was 67 compared to 61 among male patients (*p* <.05). The median resilience score among patients who received more than 9 years of education was 67 compared to 61 among those with less than 9 years of education (*p* <.05). Patients employed reported a median resilience score of 68 compared to 65 among those retired or unemployed (*p* <.05). Participants who partly knew diagnosis reported a median resilience score of 59.5 compared to 64 or 68 among those who knew nothing or were fully aware of the diagnosis (*p* <.05).

### Relationships between distress, resilience and coping strategies, anxiety, and depression

DT scores had a significant association with acceptance-resignation, HADS-A, and HADS-D (r=0.40-0.56, *p* <.001, **Table 2**). Resilience scores positively correlated with confrontation and avoidance (r=0.20-0.31, *p* <.001). Resilience scores negatively correlated with HADS-A, HADS-D and DT (r=−0.29- −0.43, *p* <.001).

### Factors influencing the level of psychological distress and resilience

As shown in **Table 3**, after adjusting for sex, time span after diagnosis, knowing of diagnosis, depression, acceptance-resignation and resilience, which were statistically associated with distress, patients with NET G1 (Odds Ratio (OR) =0.14, 95% CI: 0.03-0.68) and patients with anxiety (OR =1.57, 95% CI: 1.33-1.85) were more likely to report distress.

**Table 3:** Logistic regression analysis of factors influencing the level of psychological distress and resilience.

Dependent variable	Factors	OR	Lower 95%CI	Upper 95%CI	*P* Value
Distress ^a^	Stage of tumor	0.14	0.03	0.68	.015*
	HADS−A	1.57	1.33	1.85	<.001*
	Constant	2.08			.624
Anxiety ^b^	Avoidance	0.85	0.74	0.98	.026*
	Acceptance-resignation	1.47	1.26	1.71	<.001*
	Distress ^a^	5.74	2.70	12.20	<.001*
	Constant	0.09			.086
Depression ^c^	Confrontation	0.76	0.66	0.89	<.001*
	Avoidance	0.69	0.55	0.87	.001*
	Acceptance-resignation	1.76	1.39	2.21	<.001*
	Distress ^a^	3.31	1.25	8.76	.016*
	Constant	18.07			.143
Resilience ^d^	Confrontation	0.85	0.74	0.98	.023*
	Avoidance	0.71	0.58	0.09	.002*
	Acceptance-resignation	1.54	1.23	1.92	<.001*
	Constant	159.71			.025*

^a^0= DT < 4, 1= DT ≥ 4; ^b^0= HADS−A < 9, 1= HADS−A ≥ 9; ^c^0= HADS−D < 9, 1= HADS−D ≥ 9; ^d^0= CD-RISC ≥ 74, 1= CD-RISC < 50.

CD-RISC, Connor-Davidson Resilience Scale; CI: Confidence Interval; DT, Distress Thermometer; HADS-A, Hospital Anxiety and Depression Scale-Anxiety; HADS-D, Hospital Anxiety and Depression Scale-Depression; OR: Odds Ratio.

*p<.05.

After adjusting for sex, education, knowing of diagnosis, and depression, which were statistically associated with anxiety, patients who were coping disease with acceptance-resignation (OR=1.47, 95% CI: 1.26-1.71) and distress (OR=5.74, 95% CI: 2.70-12.2) had higher odds of anxiety.

After adjusting for education, resilience, and anxiety, which were statistically associated with depression, patients who were coping disease with acceptance-resignation (OR=1.76, 95% CI: 1.39-2.21) and distress (OR=3.31, 95% CI: 1.25-8.76) had higher odds of depression.

After adjusting for marriage, work-status, knowing of diagnosis, and distress, which were statistically associated with resilience, patients who were coping disease with acceptance-resignation had higher odds of the lowest quartile of resilience (OR=1.54, 95% CI: 1.23-1.92).

## Discussion

This cross-sectional study has four major findings. First, we found that psychological distress and low resilience in patients with GEP-NETs was common. Second, female patients were more likely to be distressed, as were those with NET G1, limited awareness of diagnosis, and with a time span from diagnosis of less than 3 months. Female patients were also more likely to have high resilience, as were those with higher education, employed, and unaware or fully aware of diagnosis. Third, distress and resilience positively correlated with coping strategies. Resilience negatively correlated with psychological distress. Finally, the factors associated with distress in patients with GEP-NETs were tumor grade, and anxiety. The factors associated with anxiety, depression and low resilience were acceptance-resignation.

### Psychological distress and resilience level

To our knowledge, this is the first study to focus on psychological distress and resilience in Chinese patients with GEP-NETs. In our study, the prevalence of distress, anxiety and depression in patients with GEP-NETs were 31.5%, 31%, and 17.8%, respectively. This prevalence of distress is much higher than results from other types of cancer in Chinese population, which was about 20% (30, 31). This strongly motivated the need to integrate the psychosocial domain into clinical practice in GEP-NETs. The prevalence of depression is lower than previously reported results (26%-40%) (7, 8). These differences may be explained by the complexity and high heterogeneity of GEP-NETs.

Patients with GEP-NETs in our study reported low scores of anxiety and depression which agree with results reported earlier in patients with midgut NET (10). In our study, patients reported a median score of resilience of 65, which is similar to a normative sample result reported previously from a Chinese study (65.4 ± 13.9) (27). However, the prevalence of low resilience in our study was high (25.9%). As resilience was found to be an important potential protective factor against psychological distress (16), a higher rate of low resilience may influence psychological distress management of patients.

### Distress and resilience under different demographics and medical characteristics

Previous studies showed that female cancer patients were more likely to be distressed, which was also observed in our study (32). In addition, the logistic regression analyses found that patients with NET G1 were more likely to report distress, which may be caused by the relatively long course of disease and complexity of treatment (33), limited treatment choices (34) as well as the worry about worsening of their disease (15). We also identified that patients with time span from diagnosis of less than 3 months were more likely to have distress, which is in consistent with previous reports (35).

An interesting finding of our study is that distress was related to limited awareness of diagnosis. About half of enrolled patients only knew their diagnosis partly, and they might not know the exact prognosis. Owing to the cultural tradition in China, breaking bad news and discussing death are harmful behaviors, and patients should be protected from knowing the truth, so some family members still cling to the tradition and oppose telling the true diagnosis to the patients, and most oncologists often don’t discuss the full diagnosis and prognosis of a malignant tumor with patients (11). However, patients who were fully aware of the diagnosis would be able to get more information about the disease from their oncologists. This communication can help building a good relationship between the patient and physician, which could then reduce psychological distress (11). Psycho-oncologists can promote communication among physicians, patients and family members through advocating an appropriated process of breaking bad news.

In our study, a higher level of resilience was found to be related to demographic characteristics including female sex, higher education, and employed, which is in line with previous reports (17, 18, 36). Patients with no knowledge or with fully awareness of the diagnosis were more likely to have a high level of resilience, which may due to a lower level of distress (17).

### Relationships between psychological distress, resilience and coping strategies

Our study suggested resilience in patients with GEP-NETs was positively correlated with confrontation and avoidance, and was negatively correlated with psychological distress. This finding is in consistent with previous studies conducted in other types of cancer (17, 19). Our results also showed that distress in patients with GEP-NETs was positively correlated with acceptance-resignation. In addition, logistic regression analyses found that patients who were coping disease with acceptance-resignation had higher odds of anxiety, depression and low resilience. Previous studies indicated that patients who used adaptive coping strategies experienced reduced distress, while those using non-adaptive coping strategies suffered from higher distress (16). Resilience mediates the relationship between the use of coping strategies and quality of life (16). Two recent multicentric studies evaluated resilience as a protective factor against the negative effects of NET on health-related quality of life (37, 38). Hence, we consider coping and resilience as critical elements able to alleviate distress and promote quality of life in patients with GEP-NETs, and psycho-oncologists should provide coping-strategy training and evidence-based resilience interventions for the patients.

### Study limitations

The study has several limitations. First, this study used a cross-sectional design which limits the causal relationship between the variables. Further prospective and longitudinal studies are required to validate our findings. Second, convenience sampling reduces the generalizability of our findings. Third, our study is a single-center research, and the results need to be confirmed in larger multi-center studies.

### Clinical implications

Additional attention is required in patients with GEP-NETs who showed a high level of psychological distress or a low level of resilience in response to their disease. This is particularly important because of the relatively long and stressful process of GEP-NETs treatment. Oncologists should feel confident to lead the disclosure of diagnosis and prognosis, as well as a discussion of treatment plans with patients and their family in order to alleviate patients’ distress. In particular, Chinese physicians should actively communicate with patients’ family members to enroll patients in the discussion of diagnosis, treatment and prognosis despite the traditional cultural taboo. Psycho-oncologists should also provide targeted interventions to patients in order to improve their psychological distress management and promote their psychological resilience. Our study highlights the need that psycho-oncologists should be incorporated into MDT for GEP-NETs patients.

## Conclusions

Our results showed psychological distress and low resilience were common in patients with GEP-NETs. Patients reporting distress were more likely to be female, with NET G1, with limited awareness of diagnosis, or with time span of less than 3 months. Patients reporting anxiety, depression or low resilience were more likely to use the coping strategy of acceptance-resignation. These findings warrant the integration of psychosocial domain into GEP-NETs clinical practice. Psycho-oncologists should play an important role in MDT for GEP-NETs patients.

## Data availability statement

The raw data supporting the conclusions of this article will be made available by the authors, without undue reservation.

## Ethics statement

This study was approved by Ethics Committee of Peking University Cancer Hospital (Reference number: 2017KT43). The patients/participants provided their written informed consent to participate in this study.

## Author contributions

LS contributed to the acquisition, analysis and interpretation of data and writing the initial draft. YC contributed to the interpretation of data and revision of the manuscript for important intellectual content. ML contributed to the conception and design of the study and data collection. JL contributed to data collection. LT contributed to the revision of the manuscript. All authors contributed to the article and approved the submitted version.

## Acknowledgments

We sincerely thank the patients enrolled in this study and their family members for supporting our work.

## Conflict of interest

The authors declare that the research was conducted in the absence of any commercial or financial relationships that could be construed as a potential conflict of interest.

## Publisher’s note

All claims expressed in this article are solely those of the authors and do not necessarily represent those of their affiliated organizations, or those of the publisher, the editors and the reviewers. Any product that may be evaluated in this article, or claim that may be made by its manufacturer, is not guaranteed or endorsed by the publisher.
